# Interfacial Water Is Separated from a Hydrophobic Silica Surface by a Gap of 1.2 nm

**DOI:** 10.1021/acsnano.4c05689

**Published:** 2024-07-08

**Authors:** Diana M. Arvelo, Jeffrey Comer, Jeremy Schmit, Ricardo Garcia

**Affiliations:** † Instituto de Ciencia de Materiales de Madrid, CSIC, Madrid 28049, Spain; ‡ Department of Anatomy and Physiology, Kansas State University, Manhattan, Kansas 66506, United States; § Department of Physics, Kansas State University, Manhattan, Kansas 66506, United States

**Keywords:** interfacial water, hydrophobic surfaces, hydrophobic gap, 3D AFM, silica−water interfaces, self-assembled monolayers

## Abstract

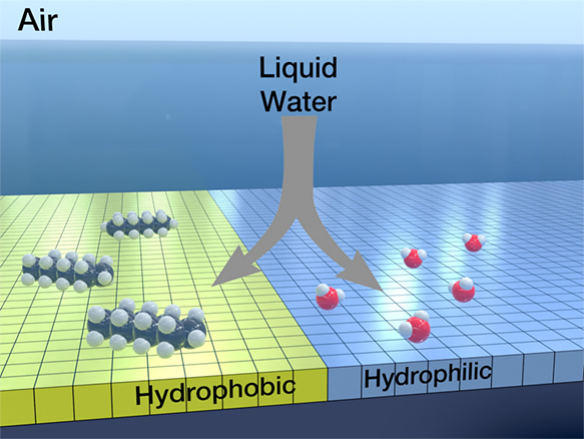

The interaction of liquid water with hydrophobic surfaces is ubiquitous in life and technology. Yet, the molecular structure of interfacial liquid water on these surfaces is not known. By using a 3D atomic force microscope, we characterize with angstrom resolution the structure of interfacial liquid water on hydrophobic and hydrophilic silica surfaces. The combination of 3D AFM images and molecular dynamics simulations reveals that next to a hydrophobic silica surface, there is a 1.2 nm region characterized by a very low density of water. In contrast, the 3D AFM images obtained of a hydrophilic silica surface reveal the presence of hydration layers next to the surface. The gap observed on hydrophobic silica surfaces is filled with two-to-three layers of straight-chain alkanes. We developed a 2D Ising model that explains the formation of a continuous hydrocarbon layer on hydrophobic silica surfaces.

The hydrophobic properties of extended surfaces are of relevance in a broad range of scientific fields, including tribology, geochemistry, molecular biology, and materials science. The interaction of water with a hydrophobic surface has implications for a variety of devices and technologies such as biosensors and water desalination devices. Consequently, many scientific contributions have been devoted to study the properties of water at a hydrophobic surface.^1−9^ However, our molecular-scale understanding of the structure of liquid water next to a smooth hydrophobic surface is far from satisfactory. Water contact angle (WCA) is the dominant technique to study the hydrophobicity of surfaces.^10^ This parameter provides a macroscopic property that does not give information on the local structure of liquid water near a solid surface. Vibrational sum frequency spectroscopy data supported the existence of water molecules with a single dangling OH bond next to the surface.^11,12^ On the other hand, X-ray reflectivity experiments supported the presence of a water density depletion layer (gap) next to hydrophobic surfaces.^3,13−15^ However, the reported thickness of the gap varied between experiments from 0.15 nm (a fraction of a water molecule)^3,13^ to 0.45 nm.^14,15^ A neutron reflectivity measurement performed in deuterated water reported a reduced water density region reaching 1.1 nm from the hydrophobic surface.^16^ However, molecular dynamics (MD) simulations did not support the existence of a gap larger than 0.2 nm.^6−8,17^ In fact, our incomplete understanding of the structure of liquid water on smooth hydrophobic surfaces comes from the limitations of the experimental methods applied to characterize those surfaces.

To resolve the above question and expand our understanding of the interaction of liquid water with a hydrophobic surface, we implement three-dimensional AFM (3D AFM) methods. Those methods provide real-space, atomic-scale resolution images of solid–liquid interfaces.^18^ To that purpose, a silicon dioxide film was functionalized with *n*-octadecyltrichlorosilane (OTS). Functionalization of silicon or silicon oxide surfaces with alkylsilane self-assembled layers (SAM) has been extensively applied in the last 25 years.^19−22^ Therefore, the functionalization protocols are well-established and routinely applied in biosensing and lithography applications. In fact, those are among the most commonly used hydrophobic surfaces in nanotechnology.

We show that on a hydrophobic silica surface, the interface has a layered structure with an interlayer distance of 0.45 nm (mean value). This distance coincides with the periodicity of straight-chain alkane layers adsorbed on a solid surface. The layered structure extends up to 1.2 nm from the surface of the OTS. Molecular dynamics simulations performed with a mixture of water and alkane molecules in the presence of the hydrophobic surface support these experimental data. In contrast, the structure of the interface measured by 3D AFM on a hydrophilic silica surface shows a considerably thinner interface (∼0.6 nm), with layers separated by 0.3 nm (mean value). The latter value is consistent with the geometry of hydration layers and with the presence of water molecules next to the silica surface.^23^

The combination of experimental and simulation findings demonstrates that liquid water is effectively separated from an extended hydrophobic surface by a gap of 1.2 nm. This gap is filled with alkane molecules that are spontaneously incorporated from the surroundings. The displacement of water by straight-chain hydrocarbons and the consequent formation of a water-depleted region are driven by hydrophobic interactions and the presence of alkanes in the environment. The presence of trace amounts of alkanes in ultrapure water and the surrounding air is unavoidable under standard conditions. Therefore, the above results represent a universal property that defines the interaction of liquid water with any extended hydrophobic surface.

## 1 Results and Discussion

**Figure 1:**
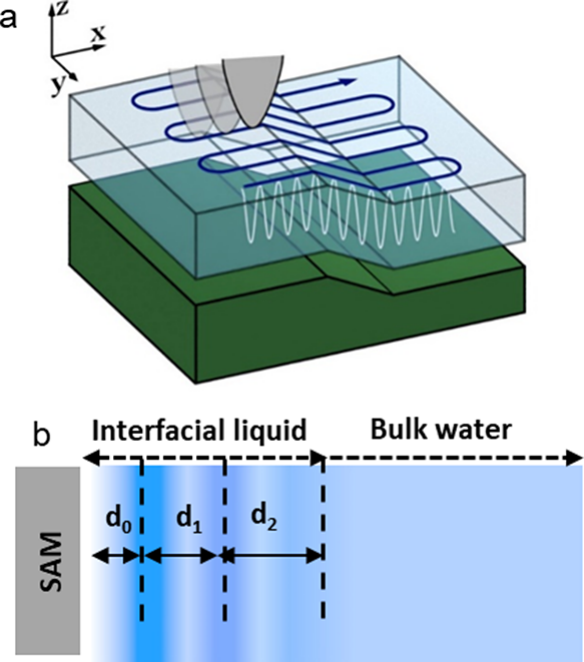
Diagrams of the 3D AFM and interfacial liquid layers. (a) Tip displacements in 3D AFM. (b) *d*_0_ is the distance between the first liquid layer and the solid surface; *d*_1_ and *d*_2_ are the distances between the liquid layers.

Figure 1a shows diagrammatically how the *xyz* displacements are performed in 3D AFM. The atomic-scale resolution features revealed by 3D AFM have been validated on a variety of crystalline surfaces immersed in aqueous solutions.^24−32^ In some cases, interpretation of 3D AFM images remains challenging.^33−36^ However, for pure water, low-molarity aqueous solutions, and organic liquids, the contrast observed in 3D AFM data can be explained in terms of the liquid density variations through the interface (Figure 1b).^33,36,37^

We apply tapping mode AFM^38^ to quantify the surface roughness and uniformity of the pristine and functionalized silicon surfaces in water before 3D AFM imaging (Figure S1, Supporting Information). The root-mean square roughness of pristine, OTS, and 3-aminopropyltriethoxysilane (APTES)-functionalized silica surfaces are, respectively, 0.5, 0.3, and 0.45 nm. Those values are consistent with the ones reported by others.^20^ Roughness values below 1 nm are characteristic of very smooth surfaces. We observe that the functionalized silica surfaces have a roughness lower than that of pristine silica surfaces. We measure the WCA on pristine and OTS and APTES-functionalized silica surfaces. The values are, respectively, 58°, 108°, and 42° (Figure S2, Supporting Information).

**Figure 2:**
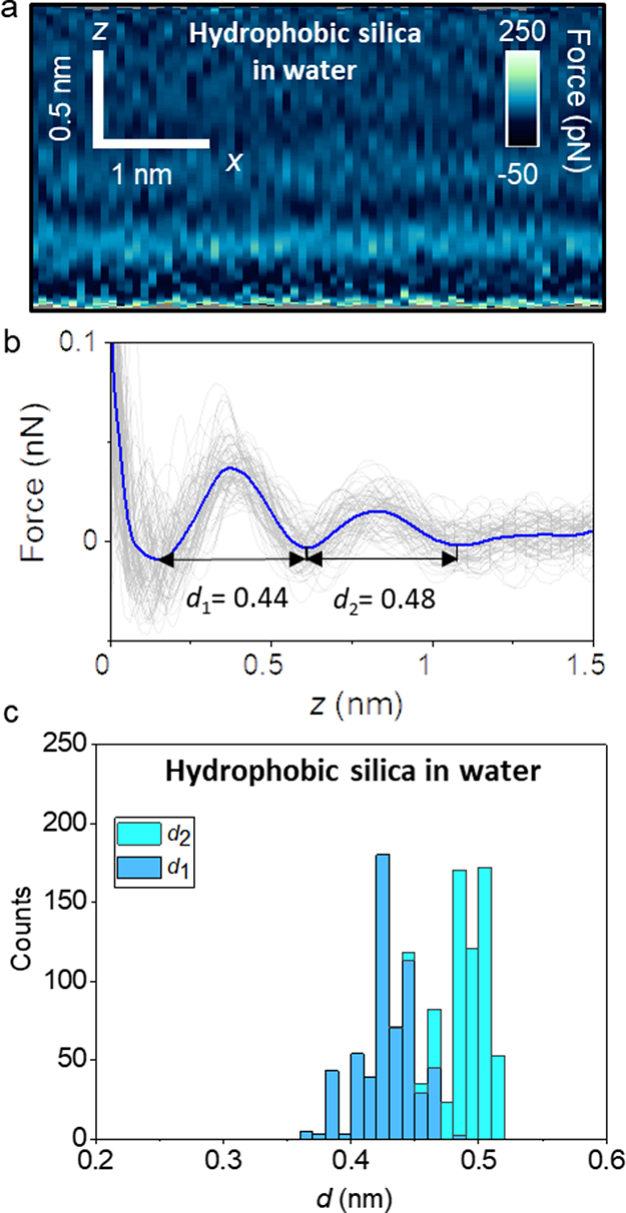
Interfacial liquid water structure on a hydrophobic silica surface (OTS). (a) 2D force maps (*x*, *z*) of an OTS–water interface (1 mM KCl). (b) Force–distance curves obtained from (a). (c) Histogram of *d*_1_ and *d*_2_ distances measured from several hydrophobic silica–water interfaces. About 600 force–distance curves are used to plot the histogram. The average force–distance curve is highlighted in blue. Individual force–distance curves are plotted in gray. Experimental parameters: *f* = 806 kHz, *k* = 9.2 N m^–1^, *Q* = 6.3, *A*_0_ = 150, and *A*_sp_ = 100 pm.

Figure 2a shows a representative 2D force map (*z–x*) of an OTS–water interface. The force map of the interface shows a layered structure characterized by the alternation of high (dark) and low (light) force regions. The layered structure extends about 1.2 nm from the outer methyl group of the OTS. At each position on the surface, we acquire a single force–distance curve *F*(*z*) perpendicular to the OTS surface (Figure 2b). Single force–distance curves are shown in gray. The *F*(*z*) plots extracted from the 2D maps show an oscillatory profile. The histograms of the distances *d*_1_ and *d*_2_ obtained from several experiments (600 force–distance curves) are shown in Figure 2c. The histograms show two distributions characterized by different median and mean values. The mean interlayer distances *d*_1_ and *d*_2_ are, respectively, 0.42 and 0.48 nm. Interfacial liquid layers typically exhibit *d*_2_ ≥ *d*_1_ because the order of the layer decreases with the distance to the solid surface.^9,34,50^ Additional 2D force maps for the OTS–water interface are shown in part 2 (Supporting Information).

The above 3D AFM results underline that the interfacial water structure on a hydrophobic surface (OTS) is characterized by interlayer distances above 0.4 nm. The periodicity of the liquid layers arises from entropic effects associated with the molecular packing of the liquid.^34,39^ The average diameter of a water molecule is 0.28 nm. Therefore, the measured values >0.4 nm are at odds with the presence of water (hydration) layers at the interface. In fact, the interlayer distance of hydration layers, as determined by different methods such 3D AFM,^18,39^ X-ray reflectivity,^41^ and MD simulations,^6,7,42,43^ ranges between 0.26 and 0.35 nm.

To identify the chemical species present at the OTS–water interfaces, we study first the interfacial structure of alkane liquids on the OTS surfaces. Based on the considerable number of studies which reported the spontaneous adsorption of alkanes from air onto graphite,^44−47^ graphene,^48−50^ van der Waals materials,^50,51^ and other surfaces,^52,53^ we hypothesize that hydrocarbons from the surroundings might be incorporated into liquid water.

**Figure 3:**
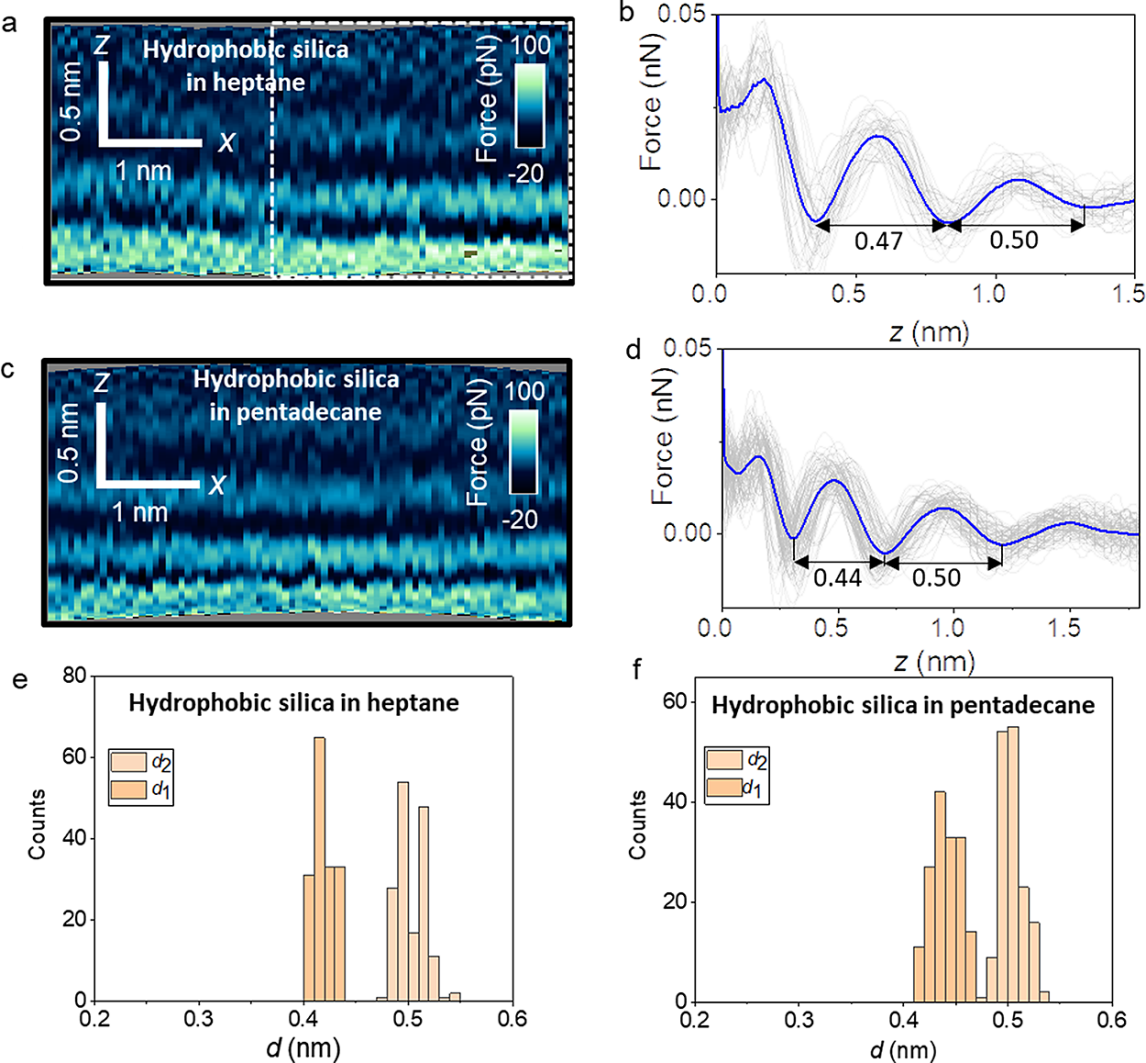
Interfacial layer structure of organic liquids on hydrophobic silica surfaces. (a) 2D force maps (*x*, *z*) of a hydrophobic silica–heptane interface. (b) Force–distance curves obtained from the rectangle marked in panel (a). (c) 2D force maps (*x*, *z*) of a hydrophobic silica–pentadecane interface. (d) Force–distance curves obtained from panel (c). (e) Histogram of *d*_1_ and *d*_2_ distances measured on hydrophobic silica surfaces in heptane. (f) Histogram of *d*_1_ and *d*_2_ distances measured on hydrophobic silica surfaces in pentadecane. The average force–distance curve is highlighted in blue in (b) and (d). Individual force–distance curves are plotted in gray in (b) and (d). Experimental parameters for heptane (pentadecane): *f* = 590,776 (476,235) kHz, *k* = 8.2 N m^–1^, *Q* = 5.4 (4.6), *A*_0_ = 100 pm, and *A*_sp_ = 60 pm.

Figure 3a–d shows the 2D maps and force–distance curves obtained by immersing the OTS surfaces in two organic liquids, heptane and pentadecane. Heptane and pentadecane molecules are examples of short and long straight-chain alkanes found in the contingent of volatile organic compounds often present in indoor air.^56,57^ For both organic liquids, the interlayer distances are in the range of 0.42–0.5 nm. The average chain lengths for heptane and pentadecane are, respectively, 0.76 and 1.8 nm. Therefore, from the above interlayer distance values, we infer that the alkanes align mostly parallel to the surface of the OTS, yielding layered structures with little dependence on alkane chain length. Figure 3e,f shows the histograms for *d*_1_ and *d*_2_ obtained from several OTS–alkane liquid interfaces. The distributions are very narrow (≤0.02 nm) and show negligible overlaps. Those distributions are associated with *d*_1_ and *d*_2_.

**Figure 4:**
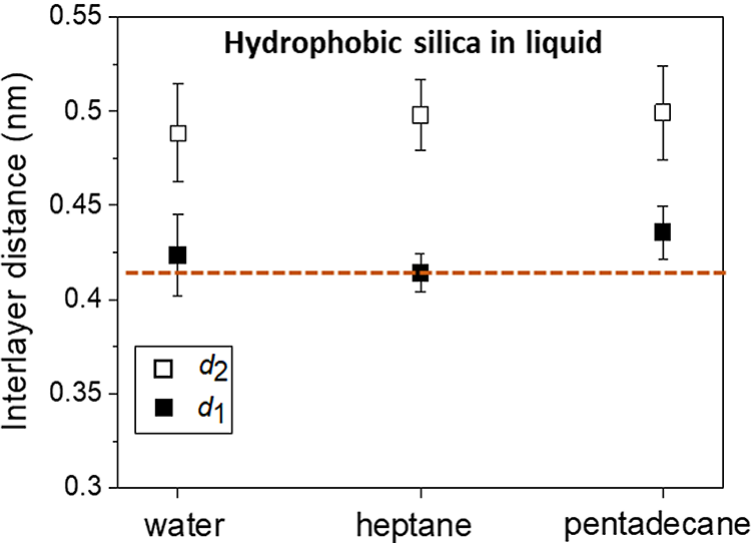
Average interlayer distance values of hydrophobic silica surfaces immersed in different liquids. The values are similar, independent of the physical properties of the liquid, water versus organic solvents. The horizontal dash line indicates the nominal diameter of a straight-chain alkane molecule (0.42 nm).

Figure 4 compares the interlayer distance values for the OTS interfaces in three solvents: water, heptane, and pentadecane. The mean values of *d*_1_ and *d*_2_ are, respectively, 0.425 and 0.48 nm (water), 0.415 and 0.49 nm, and 0.43 and 0.49 nm (Figure 3d). For each layer, the 3D AFM data show very similar values (within the error bar) for the three interfaces. Those similarities suggest that the interfacial structure observed on the surface of the OTS immersed in water is not due to water itself but might instead arise from the adsorption of straight-chain alkanes or similar molecules.

**Figure 5:**
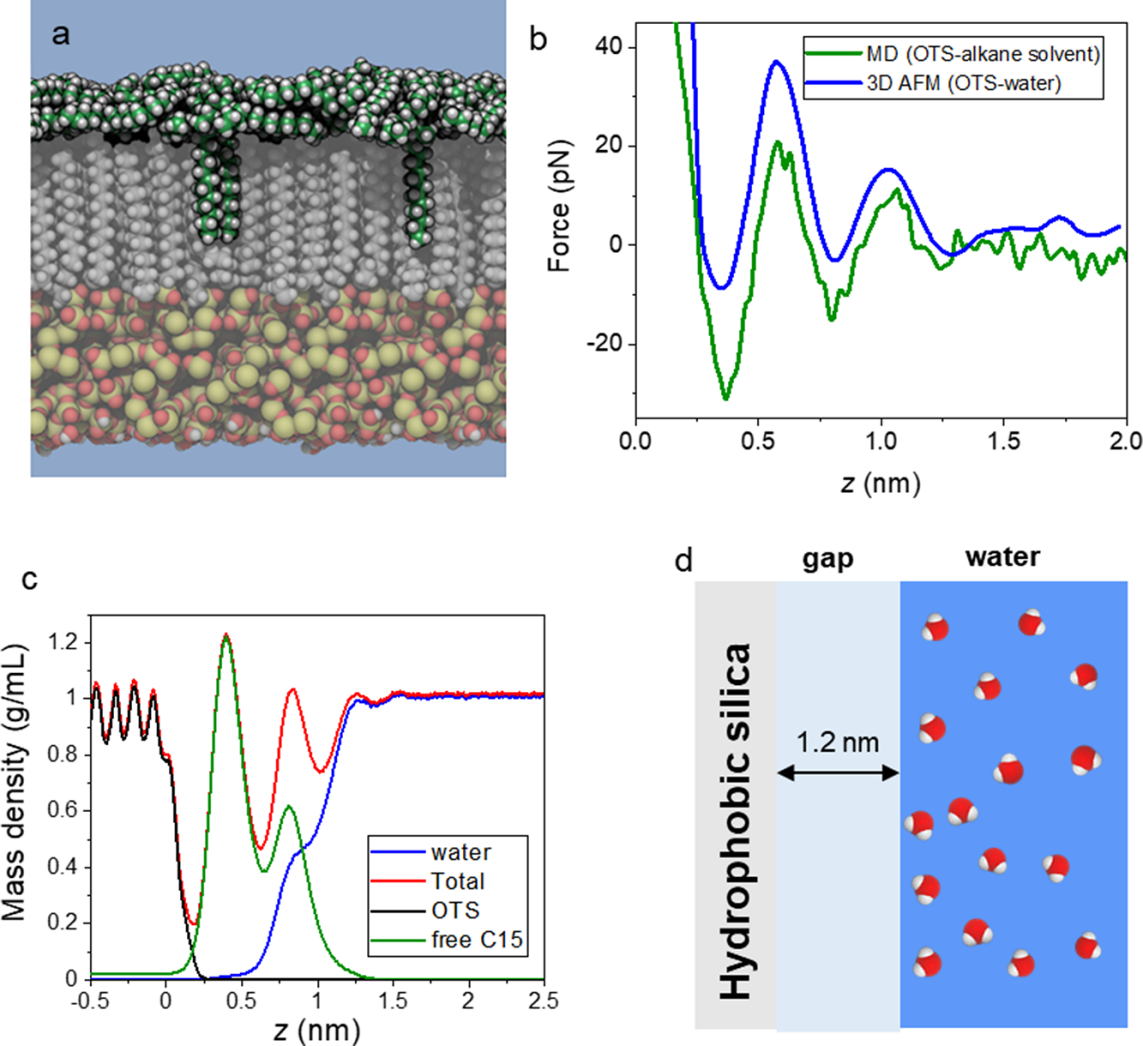
(a) MD snapshot of pentadecane molecules adsorbed on an OTS-functionalized silicon dioxide surface. C atoms are shown in green (pentadecane) or gray (OTS), while H, O, and Si atoms are shown in white, red, and yellow, respectively. The simulations included explicit water, represented as a blue background for clarity. (b) Comparison of force profiles determined by AFM for a nominally anodized OTS–water interface and by MD simulation using a model AFM tip asperity and octane solvent. The distance *z* is relative to the center of mass of the terminal CH_3_ groups of the OTS chains. The experimental force profile is shifted on the horizontal axis so that the first minimum coincides with that of the MD profile. (c) Mass density profiles for the system shown in (a). Within the OTS layer (*z* < 0), the oscillations correspond to the positions of the alkyl C atoms. At the interface (0 < *z* < 1.25 nm), the oscillations are associated with the structuring of pentadecane layers, which also coincides with the water-depleted region. The density of water reaches its bulk value 1.2 nm from the terminal C atom of the OTS chains. (d) Scheme of an OTS–water interface.

Next, we compare the force–distance curves (FDCs) measured on the OTS–water interfaces with the FDCs estimated in MD simulations. We constructed three simulation systems, one with a pristine OTS–water interface and two others with an OTS–water interface in the presence of straight-chain alkanes (heptane or pentadecane). Figure 5a shows a cross section of the OTS–water interface in the presence of alkane molecules after 600 ns of the MD simulation. Multiple layers of alkanes are visible on top of the OTS chains. We note that the structure of these layers fluctuates rapidly on the nanosecond time scale. We find that alkanes intercalate between OTS chains, preferentially at regions of lower OTS density, resulting in little dependence of the interfacial structure on the OTS functionalization density (Figure S7, Supporting Information). Consequently, there is little difference in the predicted FDCs for the OTS layers with densities of 4.0 and 5.0 chains/nm^2^ (Figure S8, Supporting Information). Most importantly, the simulations indicate that the water is expelled from the OTS surface. Figure 5b presents a comparison between the experimental and simulation FDCs. The computational force profile for the OTS–water with alkanes agrees (green) well with the corresponding experimental FDC (blue). In particular, the simulation reproduces the distance between force peaks and the number of peaks. On the other hand, the calculations using a model of an OTS–pure water interface do not even exhibit qualitative agreement with the experimental OTS–water force profile (Figure S9, Supporting Information).

Currently, the experimental 3D AFM data do not distinguish the density profiles of the different chemical species forming the interface. However, the agreement obtained between the experimental data and the MD simulation enables us to plot the total and partial mass densities across an experimental OTS–water interface. Figure 5c shows total, water, and alkane densities as a function of distance from the interface, averaged over the simulation after equilibrium was reached. The water density is well below the bulk value for distances shorter than 1 nm. In that region, the oscillatory behavior observed in the 3D AFM curves can only be associated with the layering of hydrocarbons. We conclude that water molecules are effectively expelled from the OTS surface (Figure 5d).

**Figure 6:**
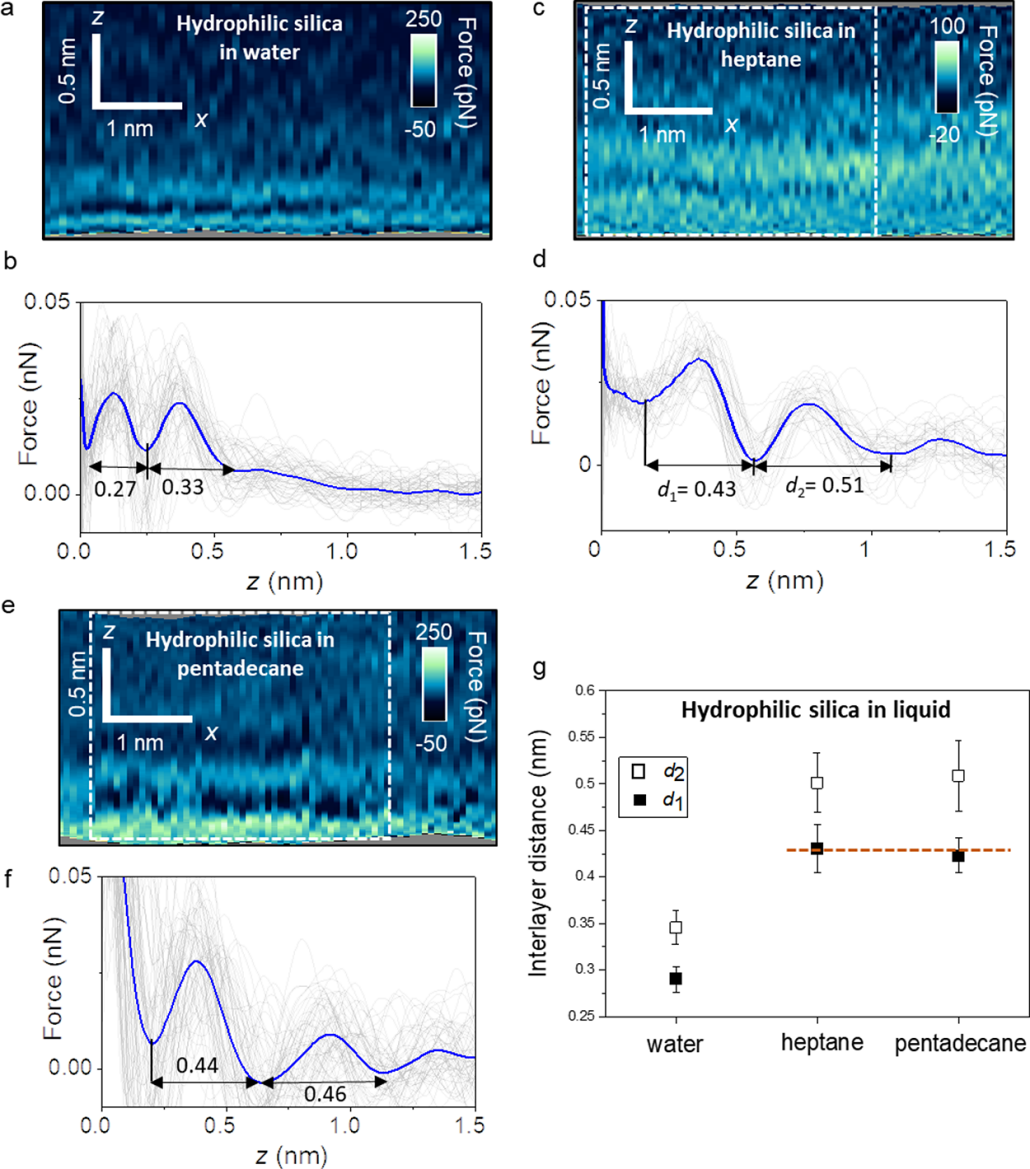
Interfacial liquid structure on a hydrophilic silica surface (APTES). (a) 2D force maps (*x*, *z*) of a hydrophilic silica–water interface (1 mM KCl). (b) Force–distance curves obtained from (a). Individual force–distance curves are plotted in gray. (c) 2D force maps of a hydrophilic silica–heptane interface. (d) Force–distance curves obtained from the rectangle marked in (c). (e) 2D force maps of a hydrophilic silica–pentadecane interface. (f) Force–distance curves obtained from the rectangle marked in (e). (g) Average interlayer distances for hydrophilic silica surfaces immersed in different liquids. The average force–distance curve is highlighted by a thick continuous line (blue) in panels (b), (d), and (f). Experimental parameters for water: *f* = 806 kHz, *k* = 9.2 N m^–1^, *Q* = 6.3, *A*_0_ = 150 pm, and *A*_sp_ = 100 pm; heptane: *f* = 478 kHz, *k* = 9.2 N m^–1^, *Q* = 5.2, *A*_0_ = 150 pm, and *A*_sp_ = 100 pm; and pentadecane: *f* = 594 kHz, *k* = 9.2 N m^–1^, *Q* = 3.5, *A*_0_ = 150 pm, and *A*_sp_ = 120 pm.

Lastly, we study the interfacial liquid water structure on a hydrophilic silicon oxide surface. For that purpose, a silicon oxide film was functionalized with APTES. An amino-terminated monolayer confers a marked hydrophilicity to silicon surfaces (Figure S2, Supporting Information). Figure 6a shows a 2D force map obtained on an APTES–water interface. Figure 6b shows the corresponding *F*(*z*) curves. The 2D maps show the presence of two solvation layers in addition to, probably, a layer of water molecules directly in contact with APTES. The interlayer distance varies between 0.3 and 0.34 nm. Those values are consistent with the average value expected for hydration layers based on theory and experiments.^6−9^ We have obtained similar results in other APTES–water experiments (Figure S4, Supporting Information).

We also study the interfacial structure of APTES with heptane and pentadecane liquids. The 2D force maps and force–distance curves (Figure 6c–f) show that the interfacial distances *d*_1_ and *d*_2_ are well above those found in water. Figure 6g summarizes the interfacial distances obtained on APTES surfaces immersed in water and alkane liquids. The data show a clear separation between the values measured in water (≤0.34 nm) and those measured in heptane and pentadecane (≥0.42 nm). They indicate that on a hydrophilic surface (APTES), the interfacial water structure is not affected by the presence of hydrocarbons in the environment. Furthermore, the values of *d*_1_ and *d*_2_ measured in APTES–heptane and pentadecane interfaces are very similar to the ones obtained for OTS in the same liquids (Figure 4). The last result indicates that the interfacial distances of the alkane liquids are independent of the hydrophilic or hydrophobic properties of the surface.

The interfacial liquid structure observed on an OTS is, perhaps surprisingly, independent of the chemical and physical properties of liquid, water (polar) versus heptane, and pentadecane (organic and nonpolar liquids). The interlayer distances are in all cases consistent with straight-chain alkane layers, which fully meets expectations for heptane and pentadecane solvents but indicates that the properties of the OTS–water interface cannot be explained by water alone. Indeed, the interfacial structure in water can be well-explained by the spontaneous migration of alkane-like contaminants from the environment to the OTS–water interface, similar to phenomena observed at aqueous interfaces of other hydrophobic materials.^40,52−55^ MD simulations corroborate this hypothesis reproducing the force–distance curves measured on an OTS–water interface only when the water includes straight-chain alkanes. The 3D AFM data show that the interfacial water structure depends on the properties of the SAM. An OTS–water interface is characterized by the presence of 2–3 layers separated by about 0.45 nm (mean value). On a hydrophilic surface (APTES), the interfacial structure is characterized by the presence of 2–3 layers separated by about 0.3 nm. The above results lead us to conclude that water is separated by a gap of about 1 nm from an extended hydrophobic surface. The gap is filled by molecules likely originating in the ambient environment with structures similar to straight-chain alkanes. The presence of hydrocarbons on hydrophobic surfaces exposed to indoor air or water should be considered to be unavoidable. Indoor air and purified water contain a trace amount of organic compounds. Among those organic compounds, linear alkanes with 15–26 carbon atoms have the highest affinity for interfaces between water and hydrophobic surfaces.^49,58,59^ The concentration of linear alkanes in air is very small (≈20 μg/m^3^),^56^ while purified water has a concentration of hydrocarbons of about 3 μg/L (3 ppb). On the other hand, numerous reports have provided evidence on the adsorption of alkanes from indoor air onto graphitic surfaces.^44−50^ On some graphite-like surfaces, those adsorbates form regular patterns (stripes) which cover large regions of the surface.^48−51^ The condensation of dissolved gas molecules in the water (mostly N_2_) was proposed to explain the formation of stripe structures on graphite surfaces.^60−62^ However, neither additional experimental data nor theoretical simulations supported the presence of structured N_2_ molecules on the graphite–water interface. The 3D AFM data were obtained by using ultrapure water and standard laboratory conditions (*T* = 300 K and ambient pressure). We plan to perform experiments as a function of pH, dissolved gases in water, and temperature to establish the environmental condition limits to obtain the above results.

The existence of a nearly complete interfacial hydrocarbon layer at aqueous interfaces of hydrocarbon materials can be theoretically justified as follows. We consider a grid of interfacial sites σ_*i*_ that can be unoccupied or occupied by single hydrocarbon molecules. The adsorption of these hydrocarbons at the interface can be modeled by using a formalism similar to the 2D Ising model
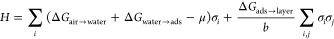
1Here, *H* is the effective Hamiltonian, Δ*G*_air→water_ is the hydration free energy of a single hydrocarbon molecule, Δ*G*_water→ads_ is the free energy of adsorption of an isolated molecule from the water phase, Δ*G*_ads→layer_ is the free energy of transferring an adsorbed molecule to the interfacial layer phase, *b* is the coordination number of a molecule in the layer phase, μ is the chemical potential of hydrocarbon molecules, and σ*_i_* ∈ {0, 1} is the occupancy of surface site *i*. The second sum is over neighboring pairs of sites ⟨*i, j*⟩; hence, Δ*G*_ads→layer_ < 0 represents an adsorbate–adsorbate attraction. In the mean-field approximation, the above Hamiltonian can be written as
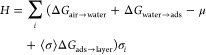
2where ⟨σ⟩ is the mean occupancy of surface sites. From this, we obtain the mean-field partition function,
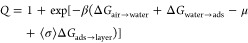
3

The partition function can then be used to compute the average site occupancy, which yields a self-consistent solution for ⟨σ⟩
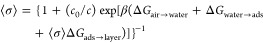
4where β = (*k*_B_*T*)^−1^. In addition, we have replaced the chemical potential with the ambient concentration in air (*c*) by
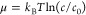
5

Eq 4 is a transcendental equation that is most easily solved by looking for intersections between the left and right sides. The left side is a straight line of unit slope, while the right side is sigmoid that approaches one as ⟨σ⟩ → ∞. The midpoint of the sigmoid shifts was left for increasing values of *c*. This means that, for large values of *c*, the only solution is ⟨σ⟩ ≅ 1, while for small values of *c*, the only solution is
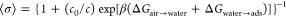
6

For intermediate values of *c*, the sigmoid rises in the physical range 0 < ⟨σ⟩ < 1, and it is possible to have three solutions. This regime represents phase coexistence with the largest and smallest solutions corresponding to the coverage of the dense and dilute phases, respectively (the middle solution is an unstable stationary state).

The model given in eq 2 treats the surface as a grid of sites that can be occupied by at most a single molecule without specifying the size of these sites; therefore, without the inclusion of additional entropy terms, the size of a state in the model is the volume per molecule in the fully occupied interfacial layer. Hence, the reference concentration *c*_0_ is the mass density of the interfacial layer phase, which can be approximated by the mass density of the pure alkane (liquid or solid).

The difference between the two sides of eq 4 is plotted in Figure S10 of the Supporting Information, with zero values representing self-consistent solutions. For very small ambient concentrations, eq 4 has only one solution, which is associated with a very low coverage. However, above a critical concentration, a high-coverage solution appears, marking the transition from adsorption of dilute, isolated molecules to nucleation and growth of a nearly complete layer. For a straight-chain alkane with 24 carbons, we estimate *c*_0_ = 0.7991 g/mL, Δ*G*_air→water_ = +5.6 kcal/mol (derived from a published Henry’s law constant^60^), Δ*G*_water→ads_ = −14.5 ± 0.3 kcal/mol, and Δ*G*_ads→layer_ = −7.6 ± 0.8 kcal/mol (see Figure S9 of the Supporting Information), giving a critical concentration for the transition to the layer phase of approximately 14 μg/m^3^.

These experiments and simulations together with the theoretical model underline that under ambient conditions, the spontaneous adsorption of hydrocarbons on hydrophobic surfaces is ubiquitous. To suppress or minimize the presence of hydrocarbons, hydrophobic–water interfaces would require electrochemical control.^63^ Those methods have not been implemented in any applications involving hydrophobic silica–water interfaces.^19−22^ In fact, the presence of trace amounts of alkanes in water (ultrapure or otherwise)^64^ or in air environments^56,57^ is unavoidable under the conditions used to conduct solid–water experiments. Therefore, the above findings should not be regarded as a contamination effect.

Based on previous results obtained on mild hydrophobic surfaces such as graphite,^9^ we propose that next to any extended hydrophobic surface, there is a 1.2 nm region of low density of water.

The formation of a water-depleted region is driven by minimization of the free energy of the system. In fact, this effect might be considered a variation of the hydrophobic interaction.^2^ In this variation, the hydrophobic surface acts as a template that favors the interaction of the solutes (straight-chain alkane molecules) dispersed in liquid water.

## 2 Conclusions

By combining three-dimensional AFM experiments and MD simulations, we demonstrate the existence of a water-depleted region next to a hydrophobic silica surface, which extends 1.2 nm into the water.

Angstrom-scale resolution images of hydrophobic silica–water interfaces reveal that the interface is characterized by the presence of two-to-three layers separated by 0.45 nm (mean value). The interlayer distance coincides with that of the adsorbed alkanes. Similar experiments performed on hydrophilic silica surfaces reveal an interface formed by one-to-two hydration layers. We conclude that straight-chain alkanes are the dominant molecular species that replace water in the gap region of a hydrophobic silica–water interface.

We develop a model of the experimental interface which involves three free energies, air–liquid, liquid–adsorbate, and adsorbate–monolayer. The model shows that the displacement of water by hydrocarbons and the consequent formation of a water-depleted region are driven by hydrophobic interactions and the presence of hydrocarbons in the environment. This behavior seems to capture the universal property of the interaction of liquid water with an extended hydrophobic surface under ambient conditions.

Many properties of alkane layers, for example, the dielectric constant, the WCA, or the vibrational modes, are similar to the properties of the OTS molecules. Those considerations might explain why the replacement of water with alkanes has not been reported before. However, there are other properties such as friction coefficients, electrical conductivities, or binding of solutes that might be significantly affected by the existence of a fluid-like alkane layer adsorbed on the hydrophobic surface.

## 3 Materials and Methods

### 3.1 3D AFM Imaging

A homemade three-dimensional AFM^40^ was implemented on a Cypher S microscope (Asylum Research, Oxford Instruments). 3D AFM is performed in amplitude modulation^38^ by exciting the cantilever at its first eigenmode. At the same time that the cantilever oscillates with respect to its equilibrium position, a sinusoidal signal is applied to the *z*-piezo position to modify the relative *z*-distance between the sample and the tip. We have used *z*-piezo displacements with amplitudes of 2.0 nm and a period (frequency) of 10 ms (100 Hz). The *z*-piezo signal is synchronized with the *xy*-displacements in such a way that for each *xy*-position on the surface of the material, the tip performs a single and complete *z*-cycle. The *z*-data are read out every 10.24 μs and stored in 512 pixels (256 pixels half cycle). Each *xy*-plane of the 3D map contains 80 × 64 pixels. Hence, the total time required to acquire such a 3D AFM image is 52 s. Additional details on the experimental methods are found in the Supporting Information.

### 3.2 Molecular Dynamics Methods

Molecular dynamics simulations were performed using NAMD 2.14^65^ using hydrogen mass repartitioning^66^ and a 4-fs time step, with the temperature and pressure maintained at 295 K and 1 atm using the Langevin thermostat and Langevin piston barostat, respectively.^67^ Alkyl groups were represented with the CHARMM General Force Field,^68^ while amorphous SiO_2_ was represented using the CHARMM-compatible parameters of Emami et al.^69^ Water used the TIP3P model of CHARMM. The force profiles were efficiently estimated by applying the adaptive biasing force (ABF) method to the distance between an AFM tip asperity and an OTS surface. The SiO_2_ AFM tip asperity model was created as described in Figure S11 of the Supporting Information. FDCs were calculated for four different atomistic simulation systems, each including a patch of amorphous SiO_2_ conjugated with an OTS at different chain densities (4.0 or 5.0 chains/nm^2^) in different solvents (water, pentadecane, octane, and decane). Octane and decane exhibited faster diffusion than the heavier alkanes, allowing the FDCs to converge on an accessible time scale. The FDC in Figure 5b is derived from more than 30 μs of simulated time. The complete systems had a mean size of 4.05 nm × 4.18 nm × 14 nm and were periodic along all three axes. Larger scale simulations, such as that shown in Figure 5a, were performed with an 8.10 × 8.36 nm OTS surface, created by duplicating the smaller system in the *x* and *y* directions. The simulation protocols and construction of the simulation models are described in greater detail in the Supporting Information.

### 3.3 Hydrophobic and Hydrophilic Silica Surfaces

The formation of self-assembled monolayers on surfaces involves two steps, the chemisorption of the molecules and the spontaneous organization of ordered domains.^70−74^ The hydrophobicity and hydrophilicity of the silica surfaces were controlled by functionalization with self-assembled monolayers, respectively, OTS (hydrophobic) and APTES (hydrophilic). Details on the preparation of the silica surfaces are provided in the Supporting Information.

## Supporting Information Available

Methods: protocol to acquire images and force–distance curves in 3D AFM; functionalization of the silica surfaces; details of MD and force-field codes; AFM tip model; thermodynamics of alkane adsorption; Figures S1: AFM images of the relevant surfaces; Figure S2: water contact angles; Figures S3–S4: 2D force (*x*, *z*) maps; Figure S5: FTIR spectrum; Figures S6 and S7: MD simulations alkanes on an OTS-functionalized silica surface; Figures S8 and S9: thermodynamics of alkane adsorption; Figure S10: tip asperity model; and Figure S11: MD snapshots of the assembly of alkane molecules on an OTS-functionalized silica surface (PDF)
