# Supplementary material for: Interfacial Water Is Separated from a Hydrophobic Silica Surface by a Gap of 1.2 nm
Source: ACS Nano. 2024 Jul 8;18(28):18683–92. doi: 10.1021/acsnano.4c05689 (PMC11256893; doi:10.1021/acsnano.4c05689)
Supplement: Supplementary file 1 — nn4c05689_si_001.pdf [file nn4c05689_si_001.pdf]

# Supporting Information

# Interfacial Water is separated from a hydrophobic silica surface by a gap of 1.2 nanometers

Diana M. Arvelo[1], Jeffrey Comer[2], Jeremy Schmit[3], and Ricardo Garcia[1]*

[1]Instituto de Ciencia de Materiales de Madrid, CSIC, 28049 Madrid, Spain.

[2]Department of Anatomy and Physiology, Kansas State University, Manhattan, Kansas, 66506, USA.

[3]Department of Physics, Kansas State University, Manhattan, Kansas, 66506, USA.

*Corresponding author: r.garcia@csic.es

**List of contents**

- Methods
- Figures S1 to S11
- References

## Methods

**3D AFM force-distance curves.** Force–distance curves (force curves) were computed from the dynamic observables $A$ and $\phi$, taking the average cantilever deflection $\Delta z$ as a function of the $z$-position. The $z$-range of the reconstructed force−distance curves was slightly reduced with respect to the amplitude and phase shift–distance curves (reduced by the zero-to-peak amplitude, i.e., $A_0$) because the force reconstruction process required an integration over the oscillation cycle. The AFM observables are acquired by modulating the tip–surface distance $z$ on different $x$ positions of the surface. The amplitude modulation AFM observables (phase shifts ($\phi$) and amplitudes ($A$)) have been transformed into force−distance curves by using some of the force reconstruction methods developed for amplitude modulation AFM[1-3]. To increase the signal-to-noise ratio in the force−distance curves we have calculated the value of the force by averaging the values of the observables for the different $x$ positions at the same $z$.

Silicon cantilevers with silicon tips were used for 3D-AFM imaging (ArrowUHF AuD, NanoAndMore, Germany). Cantilevers were cleaned first in a mixture (50:50 in volume) of isopropanol (99.6%, Acros Organics) and ultra-pure water (ELGA Maxima, 18.2 MΩ $cm^{-1}$), rinsed with ultrapure water and then placed in a UV-Ozone cleaner (PSD-UV3, Novascan Technologies, USA) for ≈ 15 min. A contactless method included in the software of the Cypher S was used to calibrate the microcantilevers. The oscillation of the cantilever was driven by photothermal excitation.

**Silicon oxide (silica) substrate.** Silicon oxide was cleaned with RCA protocol: 3 cleaning cycles with a solution containing 5 parts of ultrapure water (ELGA Maxima, 18.2 MΩ $cm^{-1}$ ), 1 part of

Ammonium Hydroxide solution 0.3 (w/w) (Sigma Aldrich) and 1 part of Hydrogen Peroxide Solution 0.3 (w/w) (Sigma-Aldrich) sonicating for 10 min. After that, 3 cleaning cycles were performed using ultrapure water sonicating for 5 min. Then the samples were cleaned by flushing with $N_2$. The cleaned samples were exposed to a plasma treatment: 50 W, 0.4 mbar for 30 s.

**Hydrophobic silica surfaces.** The hydrophobic silica surfaces were obtained by the functionalization with OTS monolayers. The cleaned $SiO_2$ samples were immersed in a solution of 5 mL toluene with 3 μL Trichloro(octadecyl)silane $\geq$ 90% (Sigma Aldrich) for 1 min and 45 s. After that, the samples were immersed in toluene (>99%, Sigma-Aldrich) in a sonicating bath for 10 min. They were then rinsed with water and dried by flushing with $N_2$. A tapping AFM image of the surface in water is shown in Figure S1(b).

**Preparation of hydrophilic surfaces**. The hydrophilic silica surfaces were obtained by functionalization with APTES monolayers**.** The cleaned $SiO_2$ samples were immersed in a solution of 5 μL (3-Aminopropyl)triethoxysilane (99%, Sigma Aldrich) in 25 mL ethanol (>99%, Sigma-Aldrich) for 45 minutes. Then the samples were rinsed with ethanol and water and dried with $N_2$ flush, heated at 80 degrees for 30 minutes and cooled down at room temperature before use. A tapping AFM image of the surface in water is shown in Figure S1(c).

**Pure water.** Ultrapure water with a resistivity 18.2 MΩ $cm^{-1}$ was freshly obtained before the experiments (ELGA Maxima). The water's pH value was 5.6 (Hanna Instruments HI 9024)

**Molecular dynamics methods.** Energy minimization, equilibration, and adaptive biasing force calculations were performed using NAMD 2.14. Energy minimization used the conjugate gradient method and equilibration was performed in the isothermal–isobaric ensemble using the Langevin thermostat (coupling parameter 1 $ps^{-1}$) and a Langevin piston barostat (period 400 fs, decay time 300 fs). Mass repartitioning of non-water hydrogen atoms was applied (giving them a mass of 3.0240 Da and reducing the mass of the attached heavy atom), which allowed use of a 4 fs second time step. Electrostatic interactions were calculated with the particle-mesh Ewald method, and Lennard-Jones interactions were computed with a smooth 10–12 Å cutoff. To improve sampling in the unbiased simulations, production simulations of the large silica-OTS systems were run using NAMD 3alpha6 with the option "cudaSOAIntegrate", which improved performance by about a factor 2 relative to NAMD 2.14 simulations for the same system.

**Models of alkylsilane-functionalized silica surfaces.** We created atomic models intended to mimic amorphous silicon dioxide surfaces functionalized by octadecyltrichlorosilane (OTS). A silanol-terminated amorphous silica slab periodic in the *xy*-plane was obtained from the model database of Emami et al.[4] (file name: silica_Q3_amorph_4_7OH_0pct_ion.pdb). Of the 84 silanol groups on the upper surface, 50, 67, or all 84 were (randomly) chosen to be conjugated with an 18-carbon alkyl chain, resulting in OTS chain densities of 3.0, 4.0, or 5.0 chains/$nm^2$. For each chosen silanol, the silanol oxygen and hydrogen atoms were deleted and the associated silicon atom was directly bonded to an octadecyl ($–CH_2(CH_2)_{16}CH_3$) group. The force profile calculations used this original silica-OTS model, which, for isothermal–isobaric molecular dynamics simulations in water at 295 K and 1 atm, had average dimensions of 4.05 nm × 4.18 nm × 14 nm, including different solvents (water, octane, decane, or pentadecane). However, to study interfacial

structure in unbiased simulations, we generated a larger silica-OTS model, created by tiling the original model in the $x$ and $y$ directions to form a $2 \times 2$ supercell (with average dimensions of 8.10 nm × 8.36 nm × 10.7 nm). For convenience and for consistency with the fact that the silica slab represents a microscopic or macroscopic object, silicon atoms with $|z - z_c| < 0.05$ nm (where $z_c$ is the $z$-component of the center of mass of the silica slab) were restrained to their initial $z$ coordinates with an energy constant of 1.0 kcal mol$^{-1}$ Å$^{-2}$. No restraint forces were applied in the $x$ and $y$ directions.

**Effect of alkylsilane chain density.** Previous molecular dynamics studies have shown that the structure of OTS layers in water varies dramatically with the areal density of OTS chains on the silica surface[5,6]. In the absence of free alkanes, we found results similar to these studies. For example, as shown in Figure S7, the OTS chains in our simulations exhibited chain tilt angles similar to those of Summers et al. [6], who report mean angles of 34.1° and 7.0° for OTS densities of 3.0 and 5.0 chains/nm$^2$, respectively. However, when free alkanes are included in the system with an OTS density of 3.0 chains/nm$^2$, they quickly intercalate the OTS chains, increasing the packing density of the layer and resulting in an OTS/alkane layer with a structure similar to an OTS layer of higher functionalization density. We performed the calculation of the force profile for both 4.0 and 5.0 chains/nm$^2$, yielding similar results as shown in Figure S9.

**AFM tip asperity model.** As outlined in Figure S11, we constructed a plausible model of the most extreme asperity of an AFM tip. This model was generated by a simulated annealing process that involved melting a collection of Si and O atoms under restraints to create a long column of amorphous silica, similar to protocols used in previous simulation studies to create relaxed amorphous $SiO_2$ structures[7]. These simulations used the ReaxFF parameters for Si–O parameters

of Nayir *et al.* [8]. The simulations were performed using LAMMPS[9], including a GPU-accelerated implementation of ReaxFF[10]. The initial conditions of these simulations consisted of 159 Si atoms and 318 O atoms occupying cylindrical region with the long axis along $z$ (Figure S11A). To maintain the cylindrical shape during the simulated annealing process, atoms at a distance more than 6 Å from the $z$-axis were subjected to a flat-bottom harmonic restraint. Specifically, the restraint energy was given by $E = k\,(s - s_c)^2$, where $k = 2$ kcal mol$^{-1}$ Å$^{-2}$ was the strength of the restraint, $s = (x^2 + y^2)^{1/2}$ was the cylindrical radial coordinate of an atom, and $s_c = 6$ Å was the distance at which the restraint became active. The simulations were performed with a time step of 0.25 fs and a Langevin thermostat (time constant of 200 fs). The periodic cell size was significantly larger than the atomic structure along all dimensions (3.0 × 3.0 × 10.0 nm$^3$). The temperature was set to 5000 K, far above the melting point of $SiO_2$, and linearly reduced to 295 K over $5 \times 10^6$ simulation steps or 1.25 ns of simulated time. The resulting amorphous silica structure (Figure S11B) was 5.3 nm in length, which was longer than desired, so the far end was truncated (Figure S11C). Two free $O_2$ molecules formed during the annealing process were also deleted. In the resulting structure, all Si atoms were coordinated with 3 or 4 oxygens and all oxygen atoms were coordinated with 1 or 2 silicon atoms.

Thirty-four silanol groups were created on the surface by adding SiOH groups to Si atoms with only 3 neighbors, and hydrogen to O atoms with only one neighbor. The final asperity model (Figure S11D) consisted of 86 Si atoms, 189 O atoms (155 as bulk $SiO_2$), and 34 H atoms, and was about 1.7 nm × 1.7 nm × 3.3 nm is size. The contact region at the bottom of the model consisted of two geminal silanol groups. This model was then combined with a model of an OTS-functionalized amorphous silica surface (Figure S11E) and solvated with octane (not shown). It should be noted that ReaxFF was used only to create the model structure and that all subsequent

calculations used a non-reactive force field[4] with permanent bonds, consistent with the approach used by others[6].

**Force fields.** Free alkanes and alkyl portions of the OTS chains were represented with the CHARMM General Force Field[11]. Water and $Na^+$ and $Cl^-$ ions were represented according to CHARMM standards (including nonzero Lennard-Jones interactions on TIP3P hydrogen atoms and specific Lennard-Jones parameters for $Na^+$–$Cl^-$ pairs)[12]. Amorphous $SiO_2$ was represented using the CHARMM-compatible parameters of Emami et al.[4] . As specified in this force field, silicon, bulk silica oxygen, silanol oxygen, and silanol hydrogen atoms were assigned charges of +1.1, −0.55, −0.675, and +0.4 (in units of elementary charge)[4] . Bonded parameters involving the Si–C bond were obtained from Lorenz et al[13]. For charge neutrality, the charge of silicon atoms bonded to the octadecyl groups was set to +0.825. Since the OTS–water interface is more than 1 nm from the Si–C bonds and the octadecyl chains are relatively flexible, it is expected that the results will depend little on the parameters chosen for the silica–octadecyl linkage.

**Force profiles.** The force profiles were estimated using the adaptive biasing force (ABF) method [16] as implemented in the Colvars module[17]. Namely, we used ABF to efficiently obtain the mean force as a function of $Z$, the $z$-projected distance between the center of mass of the tip asperity model and the center of mass of the terminal $CH_3$ group of all OTS chains. The ABF domain spanned from 1.4 to 3.8 nm with a grid spacing of 0.01 nm. The resulting force profiles were shifted by −1.722 nm, the $z$-displacement between the center of mass of the asperity model and the geminal silanol at its bottom extreme; hence, $Z = 0$ represented coincidence between the bottom of the asperity and the OTS–solvent interface. To represent the fact that the asperity would be attached to a larger AFM tip, orientational restraints were applied using a restraint on a Colvars

“orientation” quaternion with a force constant of 5000 kcal/mol. The force profile was calculated for four different systems, all with dimensions of 4.05 nm × 4.18 nm × 14 nm: the silica-OTS model with 5.0 OTS chains/nm$^2$ in water, octane, or decane solvents or the silica-OTS model with 5.0 OTS chains/nm$^2$ in octane solvent. Octane and decane were used instead of the longer alkanes expected since diffusion is much slower for these heavier alkanes, making convergence of the force profile likewise much slower. To accelerate convergence of the average force, the calculation was performed in 5–11 independent runs (>1 μs per run) with distinct initial random velocities. The total simulated time for each model was 32.2 μs (5.0 OTS chains/nm$^2$ in octane), 34.5 μs (4.0 OTS chains/nm$^2$ in octane), 27.3 μs (5.0 OTS chains/nm$^2$ in water), or 118. μs (5.0 OTS chains/nm$^2$ in decane).

**Mass density profiles.** The large silica-OTS model (8.10 nm × 8.36 nm) with an OTS density of 5.0 chains/nm$^2$ was pre-equilibrated (330 ns) in water and then combined with a sphere of 106 pentadecane molecules. The pentadecane sphere was created using PACKMOL[19] and inserted into the water layer at a minimum distance of 0.4 nm above the silica-OTS surface. Water molecules and ions within the pentadecane sphere were deleted. Additional water molecules and ions were added for final total of 12504 water molecules, 34 $Na^+$, and 34 $Cl^-$ ions in the solution, representing a 150 mmol/L NaCl solution. This structure then underwent 2000 steps of energy minimization and was equilibrated in the isothermal–isobaric (*NpT*) ensemble for 0.4 ns at 370 K and then 0.7 ns at 295 K (all at 1 atm of pressure). The system size was set to the average volume over the last 0.32 ns of the equilibration, after which the systems were simulated at constant volume (*NVT* ensemble) at a temperature of 295 K using NAMD 3alpha6 for 800 ns. The mass density profiles were calculated after discarding the first 100 ns of the *NVT* simulation.

**Thermodynamics of alkane adsorption to the OTS–water interface.** We have previously shown[20] that heavy straight-chain alkanes of 15–18 carbon atoms can spontaneously form monolayers at the graphene–water interface for ambient air concentrations as low as 1–1000 $\mu g/m^3$, similar to concentrations sometimes measured in indoor air[21]. Formation of this monolayer is driven by both the favorability for adsorption of isolated alkanes to this interface and favorable alkane–alkane interactions. The potential of mean force for C24 at the OTS–water interface is shown in Figure S9.

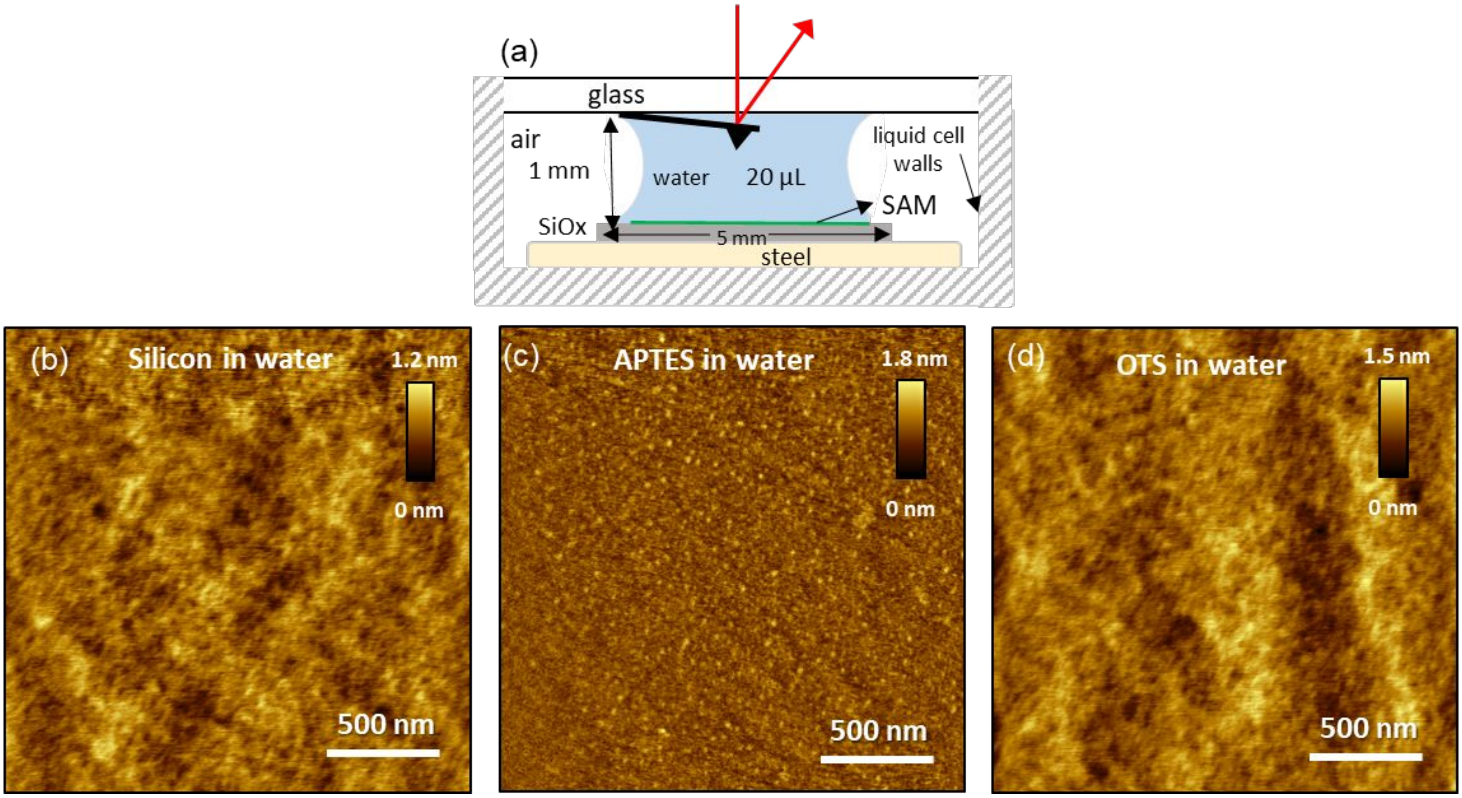


**Figure S1. Scheme liquid cell and AFM images of the functionalized silicon oxide surfaces.** (a) Scheme of the 3D AFM liquid cell. (b) Tapping mode AFM topography of an OTS functionalized silicon dioxide surface in water. (c) Tapping mode AFM topography of an APTES functionalized silicon oxide surface. (d) Tapping mode AFM topography of a silicon oxide surface in water before functionalization. Additional experimental parameters: $A_0$ = 1 nm, $A_{sp}$ = 0.8 nm; $f_1$ = 77.25 kHz, $k_1$ = 3 N m$^{-1}$, $Q_1$ = 7.

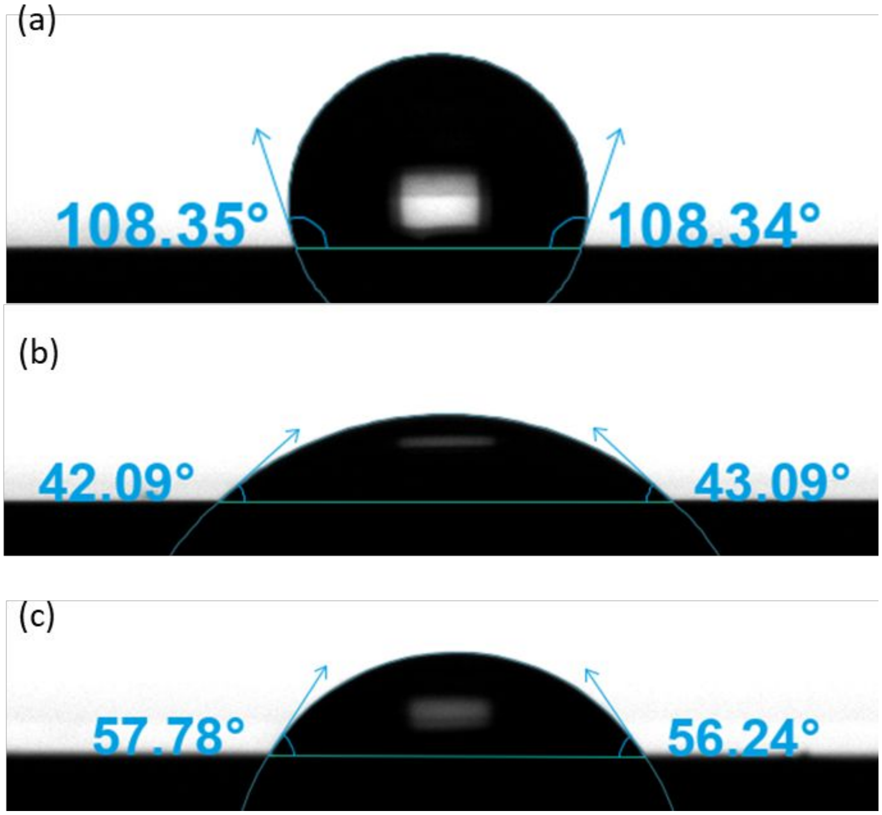


**Figure S2.** Typical water contact angle values of (a) OTS-functionalized; (b) APTES-functionalized and (c) bare silicon dioxide surfaces.

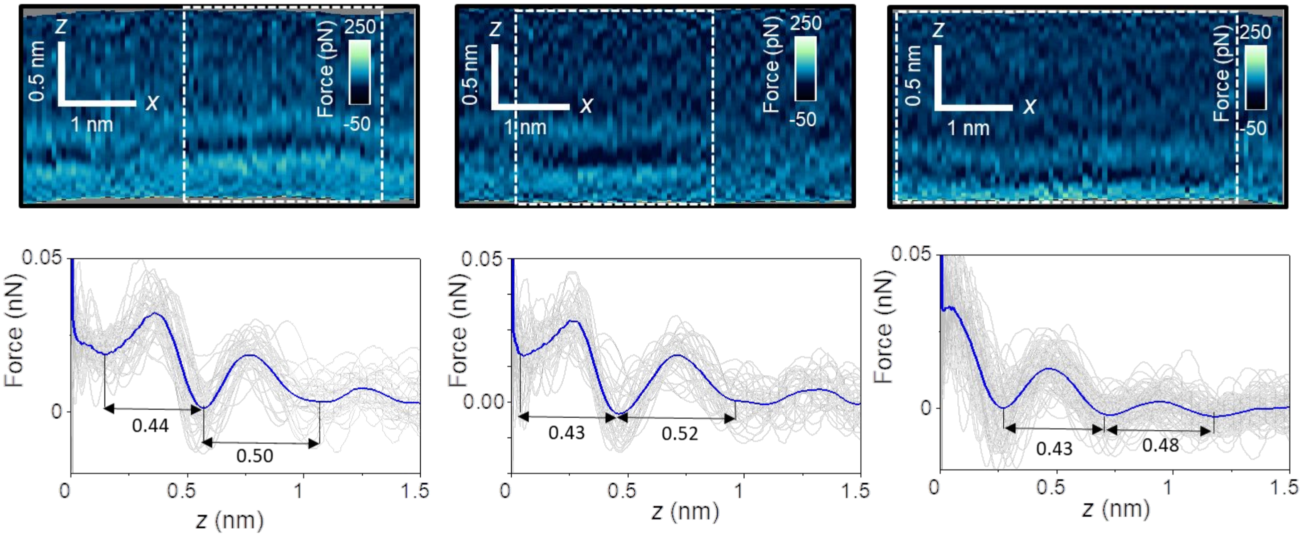


**Figure S3. Hydrophobic silica-water interfaces.** Top panels: 2D force maps (*x*, *z*) of three OTS-functionalized silica surfaces in water. Bottom panels: Force-distance curves obtained from the rectangles marked in the top panels. The average force–distance curve is highlighted by a thick continuous line (in blue). Individual force-distance curves are plotted in grey. Experimental parameters, $f_1$ = 806.613 kHz; $k_1$ = 9.2 N m$^{-1}$, $Q_1$ = 6, $A_0$ = 150 pm, $A_{sp}$ = 100 pm. Water means pure water characterized by a resistivity of 18.2 MΩ cm$^{-1}$.

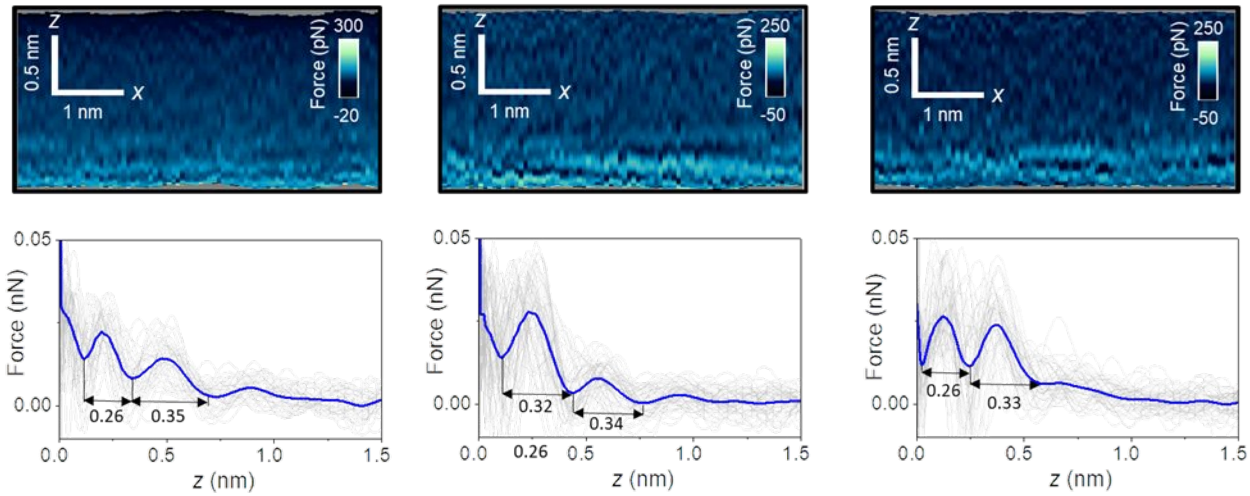


**Figure S4. Hydrophilic silica-water interfaces.** Top panels: 2D force maps (*x*, *z*) of three APTES-functionalized silica surfaces in water. Bottom panels: Force-distance curves obtained from their respective top panels. The average force–distance curve is highlighted by a thick continuous line (in blue). Individual force-distance curves are plotted in grey. Experimental parameters, $f_1$ = 806.613 kHz; $k_1$ = 9.2 N m$^{-1}$, $Q_1$ = 6, $A_0$ = 100 pm, $A_{sp}$=70 pm. Water means pure water characterized by a resistivity of 18.2 MΩ cm$^{-1}$.

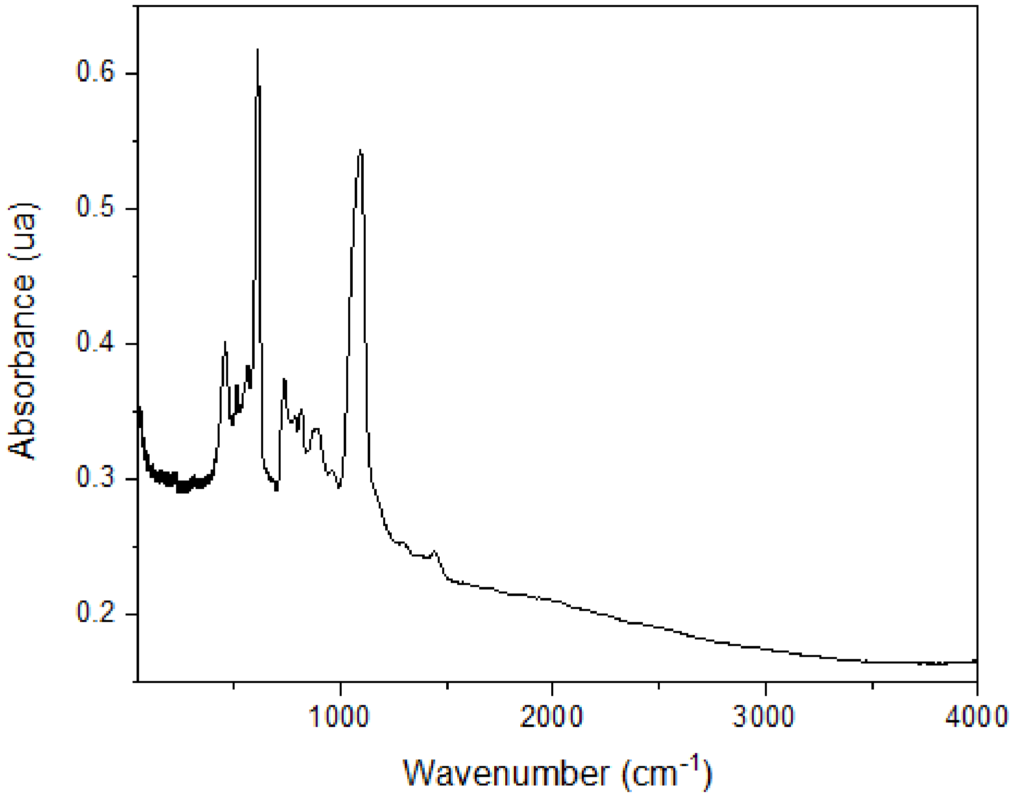


**Figure S5.** FTIR spectrum of a silicon oxide surface. The spectrum shows a frequency band at 455 cm$^{-1}$ corresponding to the Si-O rocking vibration. The peak centred at 750 cm$^{-1}$ comes from the Si-O bending vibration and the peak centred at 1090 cm$^{-1}$ corresponds to the Si-O asymmetric stretching vibrations. The presence of hydrocarbon contamination is negligible. The signal associated with hydrocarbons (CH, $CH_2$) should appear in the 2800–3000 cm$^{-1}$ range. FTIR spectrum of a 1 cm$^2$ surface of the cleaned $SiO_2$ surface. Instrument: FTIR Vertex 70V by Bruker Optics spectrometer was employed, to collect in transmission mode with 4 cm$^{-1}$ resolution under vacuum (1 mbar), at room temperature.

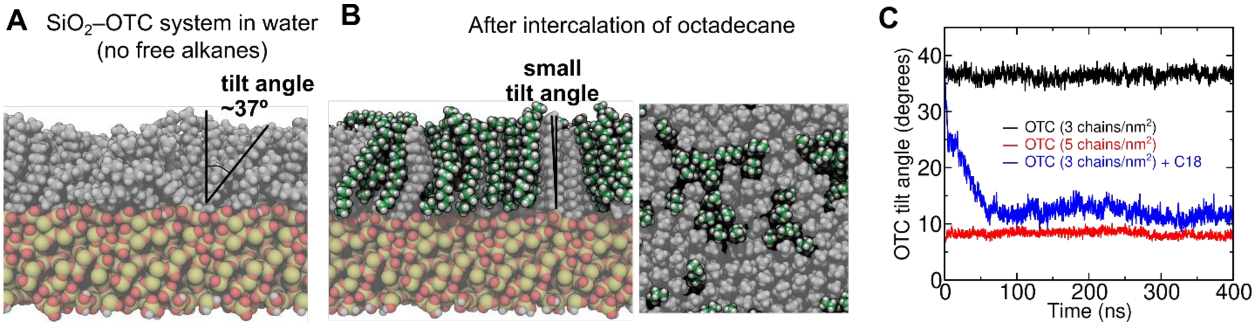


**Figure S6.** Effect of free alkanes on the structure of the alkylsilane layer. (a) Simulation snapshot showing the structure of an OTS layer with a density of 3.0 chains/nm$^2$ in water. A relatively high tilt angle is observed. (b) Simulation snapshots (left, cross section; right, from above) showing the structure of the same OTS layer in the presence of free octadecane. Octadecane intercalates the OTS layer, yielding greater packing and a smaller tilt angle. Atom colors are: H, white; alkylsilane C, grey; free octadecane C, green; O, red; Si, yellow. (c) Plot of alkylsilane tilt angle relative to the surface normal (*z*-axis) as a function of simulated time. In the presence of free octadecane (C18), the tilt angle of the alkylsilane layer with 3.0 chains/nm$^2$ becomes more like that for a layer of higher chain density due to intercalation of octadecane molecules.

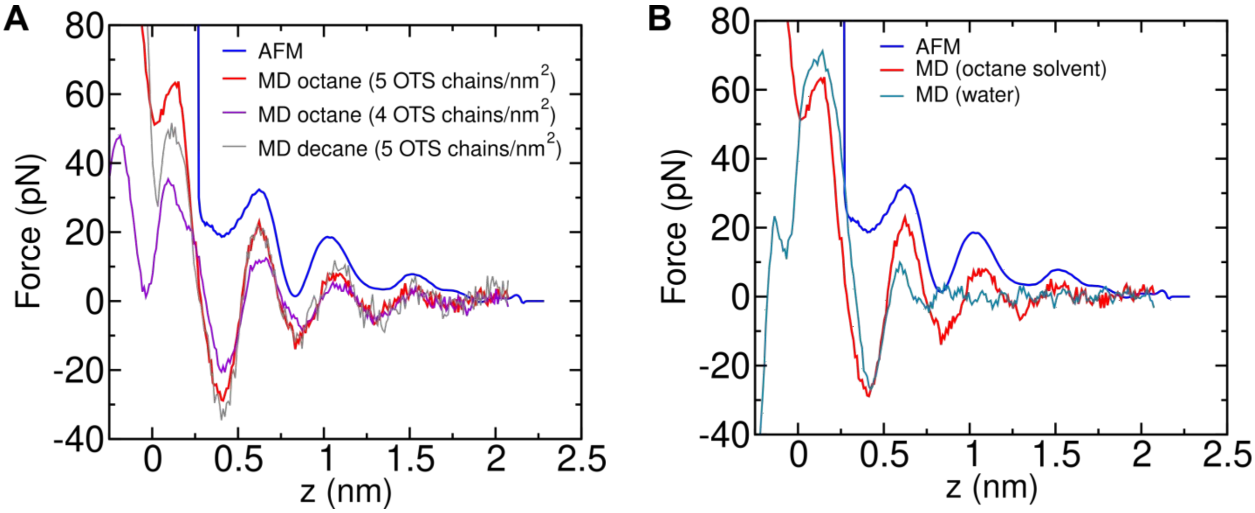


**Figure S7.** 3D AFM (experiment) force-distance curve for OTS-water interfaces and MD simulations FD curves for OTS-water and OTS-alkane solvent interfaces. (A) Different OTS chain densities (4 and 5 chains/nm$^2$) and different alkane solvents (octane and decane) yield similar features among themselves and show peak locations similar to those derived from experiments in nominally pure water. (B) A simulation model assuming a pristine OTS-water interface yields poor agreement with experiment.

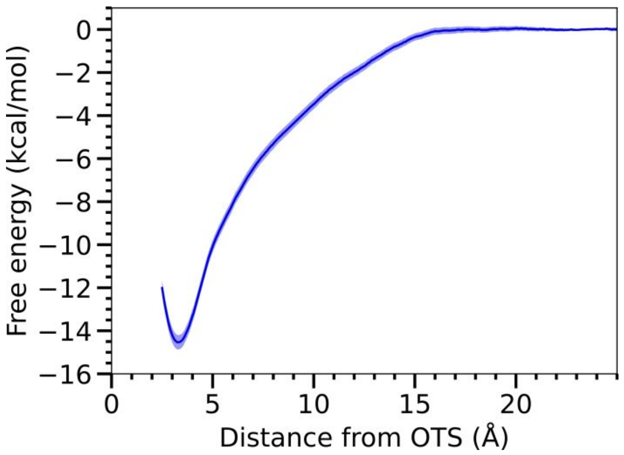


**Figure S8.** Free-energy as a function of distance between a tetracosane (C24) molecule and a pristine OTS–water interface (black).

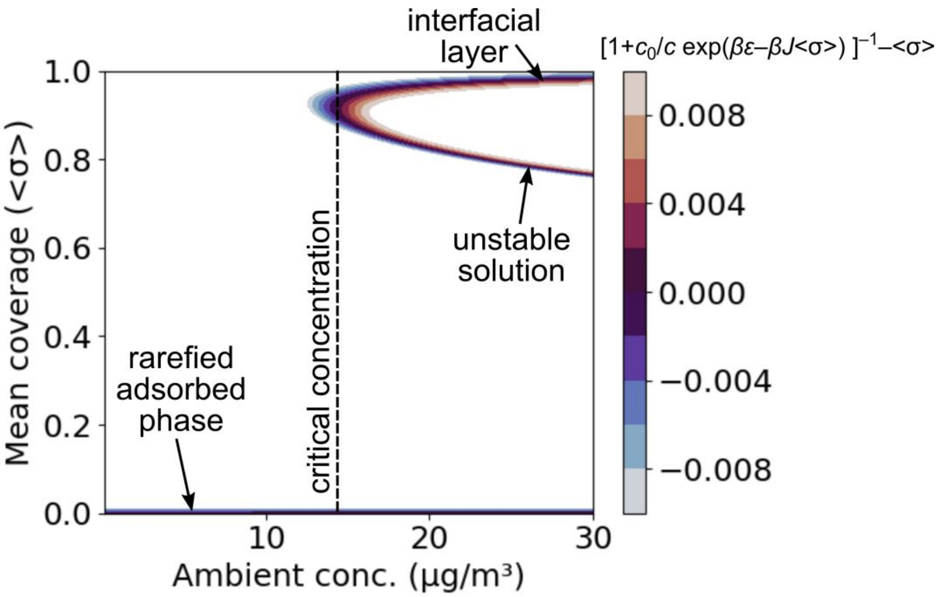


**Figure S9.** Plot of solutions to the equation $\{1 + (c_0/c) exp[\beta(\Delta G_{air\rightarrow water} + \Delta G_{water\rightarrow ads} + \langle\sigma\rangle\Delta G_{ads\rightarrow layer})]\}^{-1} - \langle\sigma\rangle = 0$ as a function of ambient concentration in air ($c$) and coverage of the OTS-water interface ($\langle\sigma\rangle$). Zero values in the plot are solutions to the 2D Ising model. Below the critical concentration (an ambient air concentration of 14 μg/m$^3$), there is only one solution of low $\langle\sigma\rangle$, representing rarefied adsorption. However, for concentrations larger than this critical concentration, there is a high coverage solution, representing a phase transition to a nearly complete interfacial C24 layer. Here, the model parameters represent $n$-tetracosane (C24), with $c_0$ = 0.7991 g/mL approximated from the experimental mass density of pure C24, $\Delta G_{air\rightarrow water}$= +5.6 kcal/mol obtained from the published Henry's law constant,[22] and $\Delta G_{water\rightarrow ads}$= −14.5 ± 0.3 kcal/mol and $\Delta G_{ads\rightarrow layer}$= −7.6 ± 0.8 kcal/mol estimated from molecular dynamics simulations (see Figure S9).

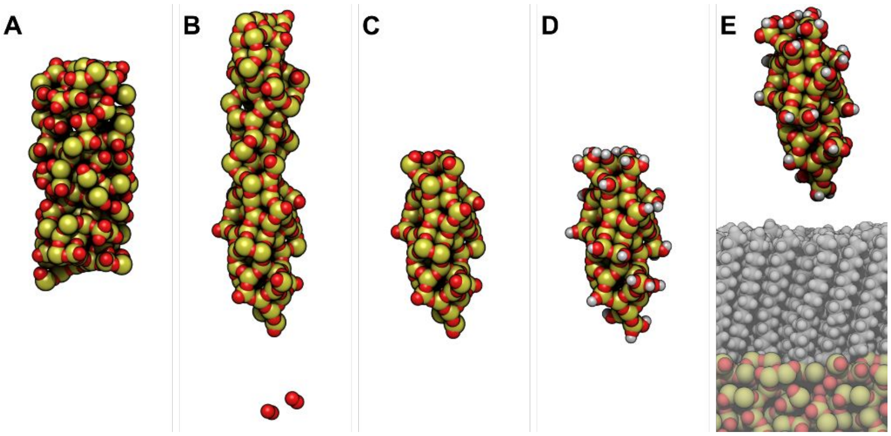


**Figure S10.** Creation of the tip asperity model. (a) Initial arrangement of Si (yellow) and O (red) atoms. (b) Amorphous $SiO_2$ structure after annealing from 5000 K using the ReaxFF framework. (c) Truncating the structure to reduce its size. (d) Capping undercoordinated atoms with silanol groups. (e) Resulting structure after combining with the silica-OTS model and equilibrating in water with the classical force field.

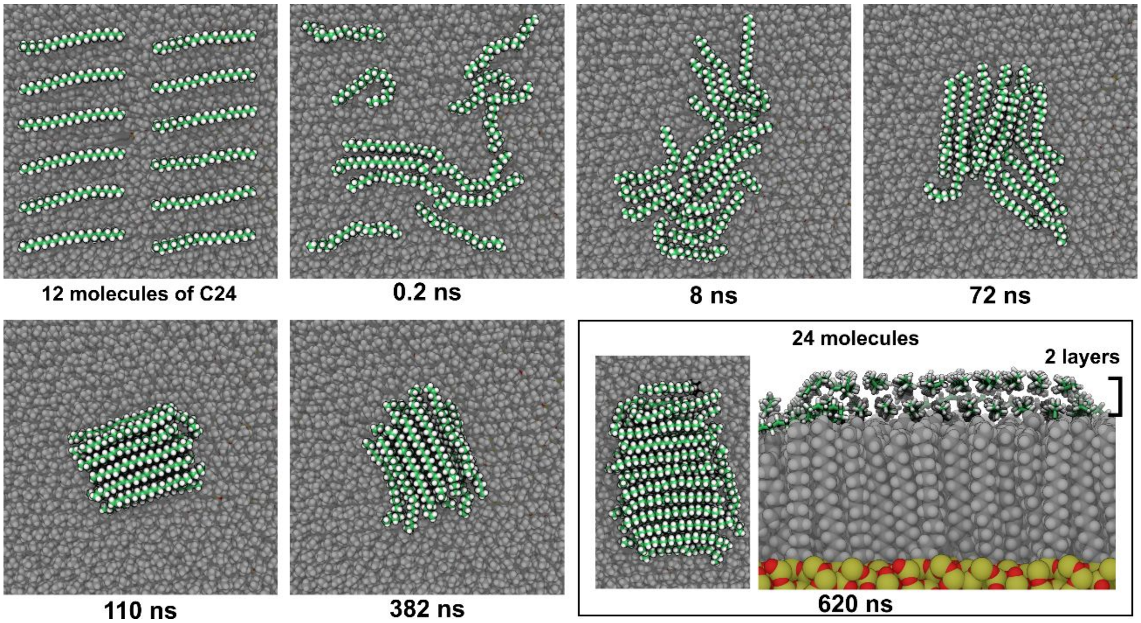


**Figure S11.** Snapshots from molecular dynamics simulations showing spontaneous assembly of twelve tetracosane (C24) molecules at the interface between water and the alkyl groups of an OTS-conjugated silica surface. C24 carbon is green, while OTS carbon is grey. Within 110 ns, a stable compact aggregate is formed. Lower right panel. Result of a similar simulation including

24 molecules. In all cases, the aggregates typically consist of two well-defined alkane layers, although the structure fluctuates somewhat on the nanosecond timescale.

**References**


1 Garcia, R. Amplitude Modulation Atomic Force Microscopy (Wiley-VCH, Weinheim, 2010).

2 Hölscher, H. Quantitative measurement of tip-sample interactions in amplitude modulation atomic force microscopy. *Appl. Phys. Lett*. **89**. 123109 (2006).

3 Payam, A.F., Martin-Jimenez, D., Garcia, R. Force reconstruction from tapping mode force microscopy experiments. *Nanotechnology* **26**, 185706 (2015).

4 Emami, F. S., Puddu, V., Berry, R. J., Varshney, V., Patwardhan, S. V., Perry, C. C., Heinz, H. Force field and a surface model database for silica to simulate interfacial properties in atomic resolution. *Chem. Mater.* **26**, 2647–2658 (2014).

5 Roscioni, O. M., Muccioli, L., Mityashin, A., Cornil, J., Zannoni, C. Structural characterization of alkylsilane and fluoroalkylsilane self-assembled monolayers on SiO2 by molecular dynamics simulations. *J. Phys. Chem. C* **120**, 14652–14662 (2016).

6 Summers, A. Z., Iacovella, C. R., Cummings, P. T., McCabe, C. Investigating alkylsilane monolayer tribology at a single-asperity contact with molecular dynamics simulation, *Langmuir* **33,** 11270–11280 (2017).

7 Litton, D. A., Garofalini, S. H. Modeling of hydrophilic wafer bonding by molecular dynamics simulations. *J. Appl. Phys.* **89**, 6013–6023 (2001).

8 Nayir, N., Van Duin, A. C., Erkoc, S. Development of the ReaxFF reactive force field for inherent point defects in the Si/silica system. *J. Phys. Chem. A* **123**, 4303–4313 (2019).

9 Plimpton, S. Fast parallel algorithms for short-range molecular dynamics. *J. Chem. Phys.* **117**, 1–19 (1995).

10 Aktulga, H. M., Fogarty, J. C., Pandit, S. A., Grama, A. Y. Parallel reactive molecular dynamics: Numerical methods and algorithmic techniques. *Parallel Comput*. **38**, 245–259 (2012).

11 Vanommeslaeghe, K., Hatcher, E., Acharya, C., Kundu, S., Zhong, S., Shim, J., Darian, E., Guvench, O., Lopes, P., Vorobyov, I., Mackerell Jr., A. D. CHARMM general force field: A force field for drug-like molecules compatible with the CHARMM all-atom additive biological force fields. *J. Comput. Chem*. **31**, 671–690 (2010).

12 Luo, Y., Roux, B. Simulation of osmotic pressure in concentrated aqueous salt solutions. J. *Phys. Chem. Lett.* **1**, 183–189 (2009).

13 Lorenz, C. D., Webb, E., Stevens, M., Chandross, M., Grest, G. Frictional dynamics of perfluorinated self-assembled monolayers on amorphous $SiO_2$. *Tribol. Lett.* **19**, 93–98 (2005).

14 Phillips, J. C. et al., Scalable molecular dynamics on CPU and GPU architectures with NAMD. *J. Comput. Chem.* **153**, 044130 (2020).

15 Feller, S. E., Zhang, Y. H., Pastor, R. W., Brooks, B. R. Constant pressure molecular dynamics simulations-the Langevin piston method. *J. Chem. Phys.* **103** 4613-4621 (1995).

16 Darden, T. A., York, D. M., Pedersen, L. G. Particle mesh Ewald: An n log n method for Ewald sums in large systems. *J. Chem. Phys.* **98**, 10089-10092 (1993).

17 Comer, J. et al., The adaptive biasing force method: everything you always wanted to know but were afraid to ask, *J. Phys. Chem. B* **119**, 1129–1151 (2015).

18 Fiorin, G., Klein, M. L., Hénin, J. Using collective variables to drive molecular dynamics simulations. *Math. Probl. Eng*. **2013**, *111*, 3345–3362.

19 Martínez, L., Andrade, R., Birgin, E. G., Martínez, J. M. Software News and Update Packmol: A Package for Building Initial Configurations for Molecular Dynamics Simulations, Journal of Computational Chemistry, **30**, 2158-2164 (2009).

20 Thakkar, R., Gajaweera, S., Comer, J. Organic contaminants and atmospheric nitrogen at the graphene–water interface: a simulation study. *Nanoscale Adv.*, **4**, 1741–1755 (2022).

21 Brown, S.K., Sim, M.R., Abramson, M.J., Gray, C.N. Concentrations of Volatile Organic Compounds in Indoor Air – *A Review. Indoor air* **4**, 123-134 (1994).

22 Sander, R. Compilation of Henry's law constants (version 4.0) for water as solvent. Atmospheric Chemistry and Physics. **15**, 4399–4981 (2015).
